# ASKA technology-based pull-down method reveals a suppressive effect of ASK1 on the inflammatory NOD-RIPK2 pathway in brown adipocytes﻿

**DOI:** 10.1038/s41598-021-01123-7

**Published:** 2021-11-10

**Authors:** Saki Takayanagi, Kengo Watanabe, Takeshi Maruyama, Motoyuki Ogawa, Kazuhiro Morishita, Mayumi Soga, Tomohisa Hatta, Tohru Natsume, Tomoya Hirano, Hiroyuki Kagechika, Kazuki Hattori, Isao Naguro, Hidenori Ichijo

**Affiliations:** 1 Laboratory of Cell Signaling, Graduate School of Pharmaceutical Sciences, The University of Tokyo, 7-3-1 Hongo, Bunkyo-ku, Tokyo, 113-0033 Japan; 2 Molecular Profiling Research Center for Drug Discovery, The National Institute of Advanced Industrial Science and Technology, 2-4-7 Aomi, Koto-ku, Tokyo, 135-0064 Japan; 3 Cellular and Molecular Biotechnology Research Institute, The National Institute of Advanced Industrial Science and Technology, 2-4-7 Aomi, Koto-ku, Tokyo, 135-0064 Japan; 4 Institute of Biomaterials and Bioengineering, Tokyo Medical and Dental University, 2-3-10 Kandasurugadai, Chiyoda-ku, Tokyo, 101-0062 Japan; 5 Present Address: Faculty of Pharmacy, Osaka Medical and Pharmaceutical University, 4-20-1 Nasahara, Takatsuki, Osaka 569-1094 Japan

**Keywords:** Kinases, Stress signalling, NOD-like receptors, Protein purification

## Abstract

Recent studies have shown that adipose tissue is an immunological organ. While inflammation in energy-storing white adipose tissues has been the focus of intense research, the regulatory mechanisms of inflammation in heat-producing brown adipose tissues remain largely unknown. We previously identified apoptosis signal-regulating kinase 1 (ASK1) as a critical regulator of brown adipocyte maturation; the PKA-ASK1-p38 axis facilitates uncoupling protein 1 (UCP1) induction cell-autonomously. Here, we show that ASK1 suppresses an innate immune pathway and contributes to maintenance of brown adipocytes. We report a novel chemical pull-down method for endogenous kinases using analog sensitive kinase allele (ASKA) technology and identify an ASK1 interactor in brown adipocytes, receptor-interacting serine/threonine-protein kinase 2 (RIPK2). ASK1 disrupts the RIPK2 signaling complex and inhibits the NOD-RIPK2 pathway to downregulate the production of inflammatory cytokines. As a potential biological significance, an in vitro model for intercellular regulation suggests that ASK1 facilitates the expression of UCP1 through the suppression of inflammatory cytokine production. In parallel to our previous report on the PKA-ASK1-p38 axis, our work raises the possibility of an auxiliary role of ASK1 in brown adipocyte maintenance through neutralizing the thermogenesis-suppressive effect of the NOD-RIPK2 pathway.

## Introduction

Growing evidence suggests that adipose tissue is an immunological organ. While adipose tissue has long been merely regarded as a lipid-storing organ, it is now widely recognized that adipose tissue expresses various receptors for cytokines and chemokines and responds to proinflammatory mediators secreted by itself^1,2^. Physiologically, low-grade chronic inflammation is observed under obesity and is strongly implicated in the onset and development of obesity-related diseases such as type 2 diabetes and cardiovascular disease^3^. Therefore, controlling inflammatory signaling in adipose tissue would be a potential target to combat obesity and obesity-induced diseases.

Adipose tissues in mammals can be classified into two types: white adipose tissue (WAT) and brown adipose tissue (BAT). While the major function of white adipocytes is to store excess energy as triglycerides, brown adipocytes uniquely express uncoupling protein 1 (UCP1), which is a principal contributor to its unique function in nonshivering thermogenesis^4,5^. BAT is less susceptible to inflammation than WAT, but sustained overnutrition ultimately induces a proinflammatory environment in BAT and results in impaired thermogenic machinery of brown adipocytes^6^. BAT from diet-induced obese mice showed increased infiltration of immune cells, as well as upregulation of proinflammatory cytokines^7^. Cold-induced UCP1 induction was suppressed in adipose tissue from obese mice^8^. Hence, these recent studies suggest that obesity-induced inflammation leads to dysfunction of brown adipocytes through the reduction of UCP1 and other thermogenic markers. However, the regulatory mechanisms of inflammation in brown adipocytes remain largely obscure.

The NOD-RIPK2 pathway plays a crucial role in host defense against bacterial infection and is associated with the onset of autoimmune disorders^9^. In a cell under bacterial infection, intracellular pattern recognition receptors sense the peptidoglycan derivatives of bacterial cell wall; that is, nucleotide-binding oligomerization domain 1 (NOD1) and NOD2 recognize meso-diaminopimelic acid (DAP) and muramyl dipeptide (MDP), respectively. Upon ligand binding, NODs oligomerize through the caspase recruitment domain (CARD) and induce further oligomerization of another CARD-containing protein, receptor-interacting serine/threonine-protein kinase 2 (RIPK2). Oligomerized RIPK2 is K63-polyubiquitinated by X-linked inhibitor of apoptosis protein (XIAP), linear ubiquitin chain assembly complex (LUBAC), and other E3 ligases and further recruits its downstream effectors, including TGF-beta activated kinase 1 (TAK1)/TAK1 binding protein (TAB) complex and nuclear factor of kappa B (NF-κB) essential modulator (NEMO) complex. Consequently, the c-jun N-terminal kinase (JNK), p38 mitogen-activated protein kinase (MAPK) and NF-κB pathways are activated, leading to the induction of proinflammatory cytokines^10^.

In addition to the role in immune cells, the NOD-RIPK2 pathway is implicated in adipose inflammation and affects the physiology of adipocytes. In adipocytes, pattern recognition receptors including NOD1 are considered to be activated by bacterial fragments translocated from gut microbiota^11^, which is augmented under obesity^12^. NOD1 activation in white adipocytes induces insulin resistance and lipolysis^13–15^ and suppresses adipocyte differentiation with attenuated expression of adipocyte markers and lipid accumulation^16^. Moreover, NOD1 activation in brown adipocytes leads to suppression of brown adipocyte markers, including UCP1^17^. These lines of evidence suggest that the inflammatory NOD-RIPK2 pathway in adipocytes suppresses the differentiation of adipocytes.

We have previously reported apoptosis signal-regulating kinase 1 (ASK1)^18^ as a critical regulator of thermogenesis; under β-adrenergic receptor stimulation, protein kinase A (PKA) activates the ASK1-p38 MAPK axis to induce brown adipocyte-specific genes^19,20^. Here, we show that ASK1 suppresses the NOD-RIPK2 pathway in brown adipocytes. We report an analog sensitive kinase allele (ASKA) technology-based pull-down mass spectrometry (MS) method and identify RIPK2 as a novel interactor of ASK1 in brown adipocytes. ASK1 interferes with the NOD-RIPK2 pathway by inhibiting the activation of the RIPK2 signaling complex. As a potential biological significance, our in vitro model for intercellular thermogenic regulation implies that the suppressive function of ASK1 in the NOD-RIPK2 pathway positively contributes to the maintenance of thermogenic function in BAT under inflammation, which suggests a complementary role to the ASK1’s function as a positive regulator of BAT thermogenesis via PKA-ASK1-p38 axis. This work demonstrates an example application of our novel chemical pull-down method and reveals the multifaceted finetuning role of ASK1 in brown adipocytes.

## Results

### ASKA technology-based pull-down MS method identified RIPK2 as an interactor of ASK1

**Figure 1:**
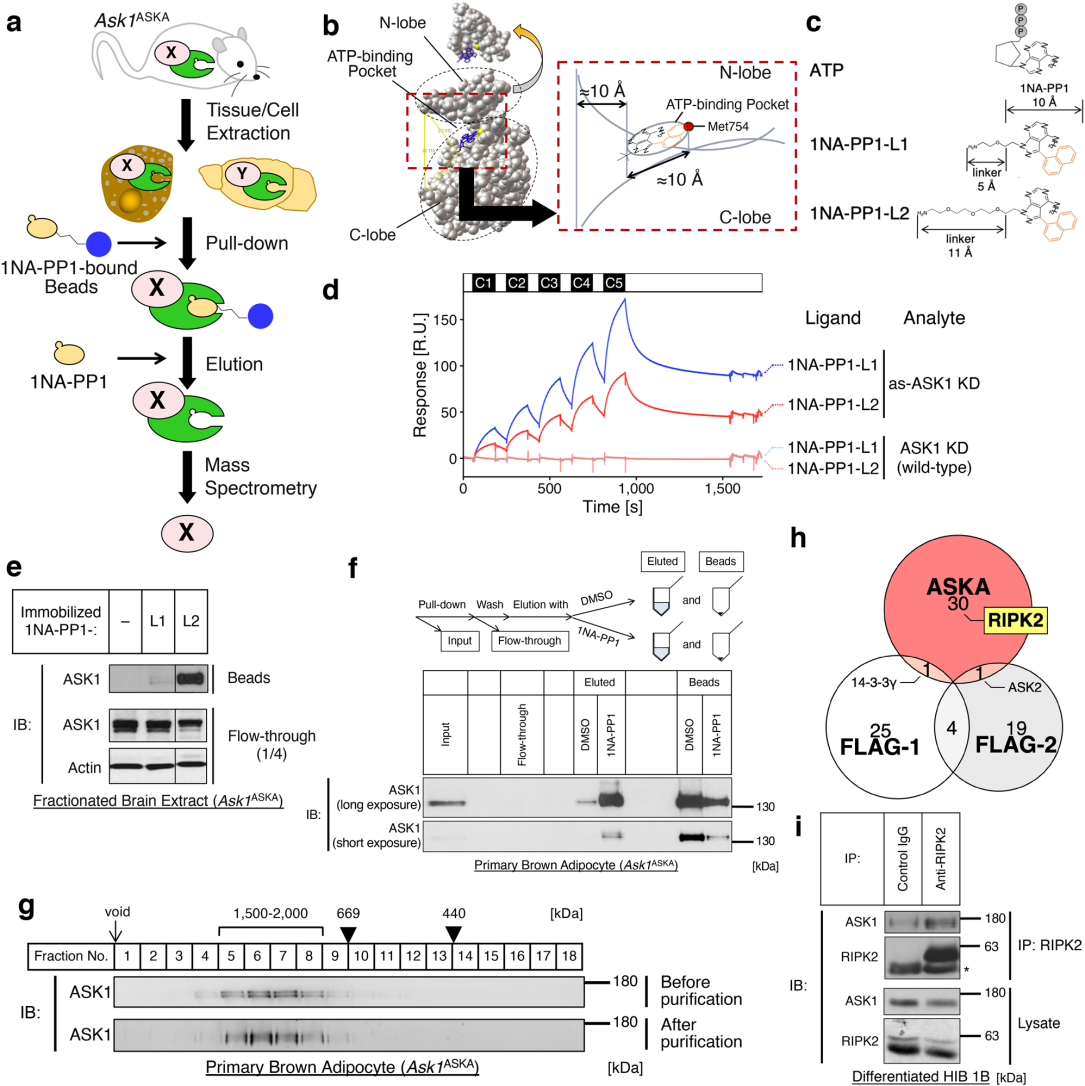
The ASKA technology-based pull-down MS method identified RIPK2 as an interactor of ASK1. (**a**) Overview of the ASKA pull-down MS method. The endogenous as-kinase signalosome was pulled down by incubating tissue/cell extracts from ASKA knock-in mice with 1NA-PP1-bound carrier beads, eluted by adding excess free 1NA-PP1, and subjected to mass spectrometry analysis. X and Y indicate unidentified components of the signalosome. (**b**) ATP-binding pocket of the ASK1 kinase domain (PDB: 2CLQ)^24^. (**c**) Chemical structures of 1NA-PP1 derivatives (1NA-PP1-Lx, x ∈ {1, 2}). *1NA-PP1* a bulky analog of ATP. (**d**) Binding affinity between the as-ASK1 kinase domain (KD) and 1NA-PP1-Lx. Setting 1NA-PP1-Lx as the ligand, the surface plasmon resonance signal was measured under a series of concentrations of the ASK1 KD analytes (white rectangle period: buffer, black rectangle period C1: 0.04 µM, C2: 0.06 µM, C3: 0.09 µM, C4: 0.13 µM, C5: 0.20 µM). *R.U.* resonance unit. (**e**) Pull-down ability of 1NA-PP1-Lx-immobilized beads for endogenous as-ASK1 in tissue lysate. Fractionated brain extracts from *Ask1*^ASKA^ knock-in mice were incubated with 1NA-PP1-Lx-immobilized beads, and the incubated beads and flow-through samples were subjected to immunoblotting (IB). Note that superfluous lanes were digitally eliminated from blot images as indicated by black lines and the uncropped images are presented in Supplementary Data. (**f**) Specific elution of as-ASK1 with 1NA-PP1. as-ASK1 was pulled down from the cell lysate of *Ask1*^ASKA^ knock-in mouse-derived primary brown adipocytes using 1NA-PP1-L2-immobilized beads and eluted with excess free 1NA-PP1. *DMSO* solvent of 1NA-PP1, a negative control. (**g**) Size of the as-ASK1 signalosome before and after purification. Cell lysates from primary brown adipocytes of *Ask1*^ASKA^ knock-in mice before and after purification with 1NA-PP1-L2-immobilized beads and 1NA-PP1 were fractionated with size exclusion chromatography. (**h**) Venn diagram of the ASK1 interactor candidates. *ASKA* ASKA pull-down MS for as-ASK1 derived from primary brown adipocytes in *Ask1*^ASKA^ knock-in mice, *FLAG-1* Flag-tag pull-down MS for Flag-ASK1 derived from overexpressed HEK293A cells^27^, *FLAG-2* Flag-tag pull-down MS for ASK1-HA-Flag derived from overexpressed HEK293 cells^28^. See Table 1 for each protein list. (**i**) Interaction between RIPK2 and ASK1 in a brown adipose cell line. Differentiated HIB 1B cells were processed for immunoprecipitation with anti-RIPK2 antibody. *IP* immunoprecipitation. *Immunoglobulin G heavy chain.

ASK1 forms a mega-Dalton complex (ASK1 signalosome) in a cell^21^. To explore unrevealed mechanisms or functions of ASK1 in BAT, we first sought to identify components of the ASK1 signalosome in brown adipocytes. ASKA technology is a kinase modification method that was originally developed to specifically inhibit a genetically modified kinase (as-kinase) with the ATP analog 1NA-PP1^22^; while bulky 1NA-PP1 cannot enter the ATP-binding pocket of wild-type kinases, the modification to substitute less bulky amino acids for residues in the hydrophobic gatekeeper region of ATP-binding pocket enables 1NA-PP1 to enter the ATP-binding pocket of as-kinase and to compete with ATP for the as-kinase. We have previously generated *Ask1*^ASKA^ knock-in mice harboring an ASKA of *Ask1* and demonstrated that primary cells from *Ask1*^ASKA^ knock-in mice showed expression and activation levels of ASK1 comparable to those from wild-type mice^23^. In this study, by leveraging the highly specific binding affinity of 1NA-PP1 to the as-kinase, we developed a chemical pull-down assay for an endogenous kinase, referred to as the “ASKA pull-down MS method” (Fig. 1a). In brief, the endogenous as-kinase signalosome was pulled down by incubating tissue/cell extracts from ASKA knock-in mice with 1NA-PP1-bound carrier beads, eluted by adding excess free 1NA-PP1, and subjected to MS analysis. To estimate the optimal linker length between 1NA-PP1 and its carrier bead, we checked the ATP-binding pocket of the ASK1 kinase domain by analyzing the previously reported crystal structure^24^ (Fig. 1b). Based on the assumed depth of the ATP-binding pocket, we synthesized two 1NA-PP1 derivatives with different linker lengths (1NA-PP1-Lx, x ∈ {1, 2}, Fig. 1c, Supplementary Note). Of note, the carrier beads we used have an approximately 20 Å linker with the *N*-hydroxysuccinimide reactive group, which cross-links with each 1NA-PP1-Lx. Using a surface plasmon resonance (SPR) assay, we confirmed the direct biophysical affinity of 1NA-PP1-Lx with the recombinant as-ASK1 kinase domain (KD) in vitro but not with wild-type ASK1 KD (Fig. 1d), validating that our pull-down strategy specifically captures as-kinase. In addition, because the analyte ASK1 KD can be dimerized in solution^24^, we modeled the bivalent analyte model, which fit our SPR data well. The dissociation constant for the first phase (*K*_D1_) of 1NA-PP1-L1 or 1NA-PP1-L2 vs. as-ASK1 KD was calculated as *K*_D1_ = 2.06 × 10^−6^ [M] or 2.23 × 10^−6^ [M], respectively, implying that this affinity is within a suitable range not only for pull-down but also for the subsequent elution step (Fig. 1a). We next compared the pull-down capacity of each 1NA-PP1 derivative for as-ASK1 in tissue lysates derived from *Ask1*^ASKA^ knock-in mice. Interestingly, while 1NA-PP1-L2-immobilized beads successfully pulled down as-ASK1 from brain samples, 1NA-PP1-L1-immobilized beads failed to capture as-ASK1 (Fig. 1e). This discrepancy between the direct biophysical affinity and the pull-down capacity of 1NA-PP1-L1 probably stems from the accessibility of 1NA-PP1-L1 to the ATP-binding pocket; since the entry of the 1NA-PP1 group to the ATP-binding pocket of as-ASK1 may be hindered by physical repulsion of the carrier bead with the components of the signalosome or with C/N-terminal regions of ASK1, 1NA-PP1-L1 would be too short to capture as-ASK1 in the signalosome. Hence, we adopted 1NA-PP1-L2 for the following pull-down experiments. In the final step of the purification procedures, as-ASK1 was effectively eluted from the incubated 1NA-PP1-L2-immobilized carrier beads by competitive elution with free 1NA-PP1 (Fig. 1f). To examine whether as-ASK1 maintains an intact signalosome after the series of purification procedures, we performed a size exclusion chromatography analysis and compared the as-ASK1 signalosomes before and after purification. After purification from primary brown adipocytes of *Ask1*^ASKA^ knock-in mice, as-ASK1 was observed in the same fractions of as-ASK1 before purification (Fig. 1g), suggesting that the as-ASK1 signalosome is kept intact throughout all purification steps. Therefore, as-ASK1 signalosomes purified from primary brown adipocytes of *Ask1*^ASKA^ knock-in mice were subjected to MS analysis. Comparing the MS results of 1NA-PP1-eluted samples with that of the DMSO-eluted negative control, 32 candidates were identified as interactors of ASK1 in brown adipocytes (Table 1). Among them, previously reported ASK1 interactors were included, such as ASK2 (also known as MAP3K6), a member of the ASK family^25^, and 14-3-3γ (also known as YWHAG), a negative regulator of ASK1^26^. In contrast to the ASK1 interactor candidates identified by the Flag-tag pull-down MS of Flag-tagged ASK1-overexpressing HEK293 cells in previous reports^27,28^, the identified ASK1 interactor candidates were relatively unique, with 30 out of 32 candidates categorized as brown adipocyte-specific interactors (Fig. 1h, Table 1), implying that our novel method listed up brown-specific ASK1 interactor candidates. We further validated the interaction between ASK1 and an ASK1 interactor candidate with coimmunoprecipitation assay. By immunoprecipitating RIPK2, one of the brown-specific interactor candidates, the coimmunoprecipitation of endogenous ASK1 was confirmed in the brown adipose cell line HIB 1B^29^ (Fig. 1i), supporting the validation of our ASKA pull-down MS method. Of note, it might have been possible to regard RIPK2 as an artifact in our system because RIPK2 was reported to bind directly to 1NA-PP1^30^, but this data using other method suggests that RIPK2 is a true positive interactor of ASK1.

**Table 1:** List of the ASK1-interacting candidates.

ASKA	FLAG-1	FLAG-2
Acly	ARRB2	ALDH1L1
Adcy1	ATAD3A	ATAD3A
Aifm2	BCAP31	ATAD3B
Atp12a	CDC37	ATAD3C
Car3	EIF4B	CAND1
Cfb	MOGS	CDC37
Cs	TECR	CHPF2
Echs1	HADHA	CHTOP
Ercc5	HADHB	CUL3
Fabp3	HSD17B12	DECR2
Gapdh	IKBIP	FBXW11
Gcn1l1	PGAM5	HDAC6
Itgb4	PHB2	JPH1
**Map3k6**	PRMT5	KCNAB2
Mapk1	LRRC59	MAP3K15
Mettl16	PSMD3	**MAP3K6**
Myl6	RPL23	NACC1
Ntm	SGPL1	PRKAA1
Pcdhgb8	SKP1	PRKAA2
**Ripk2**	SLC25A1	PRKAB1
Rpl8	SNRPD3	PRKAG1
Slc8a1	TXN	SGPL1
Svs2	VAPA	SLC25A10
Tdrd6	VAPB	VAPB
Tldc1	YWHAB	
Ttyh1	YWHAE	
Vat1	**YWHAG**	
Vmn1r87	YWHAH	
Vps33a	YWHAQ	
Vwa3b	YWHAZ	
Xirp2		
**Ywhag**		

The ASK1 interactor candidates from ASKA pull-down MS for as-ASK1 derived from primary brown adipocytes in *Ask1*^ASKA^ knock-in mice (ASKA), Flag-tag pull-down MS for Flag-ASK1 derived from overexpressed HEK293A cells^27^ (FLAG-1) and Flag-tag pull-down MS for ASK1-HA-Flag derived from overexpressed HEK293 cells^28^ (FLAG-2). Each pull-down identified 32, 30 and 24 interactor candidates, respectively.

Proteins highlighted in bold are the molecules mentioned in the main text. See Fig. 1h.

### ASK1 inhibits the activation of the RIPK2 signaling complex

**Figure 2:**
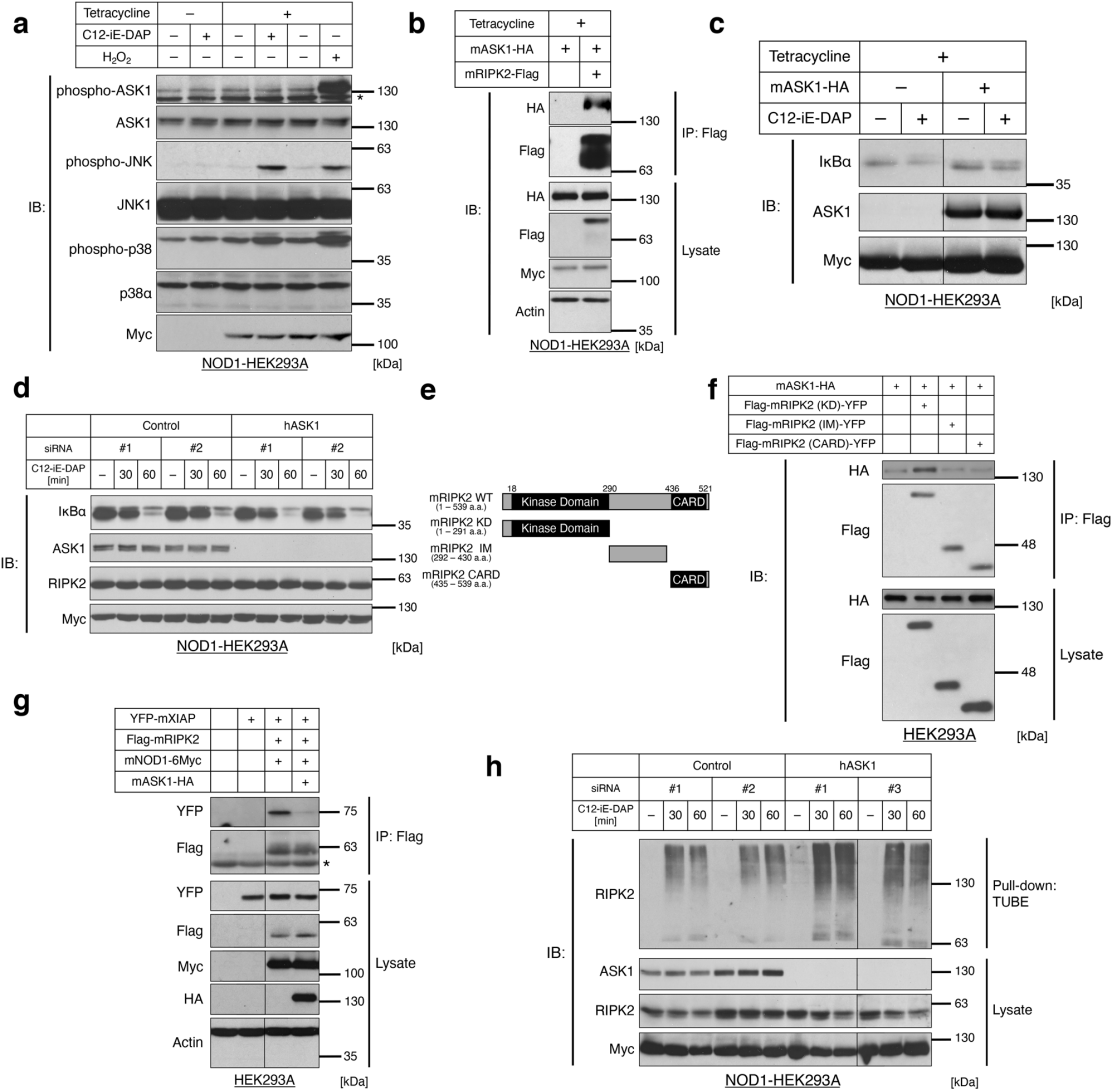
ASK1 inhibits the activation of the RIPK2 signaling complex. (**a**) Phosphorylation levels of ASK1 under NOD1 ligand treatment in NOD1-6Myc-stably expressing HEK293A cells (NOD1-HEK293A cells). NOD1-HEK293A cells were stimulated with a NOD1 ligand C12-iE-DAP (1 µg/mL, 30 min). *mNOD1* mouse NOD1, *H*_*2*_*O*_*2*_ a positive control of ASK1 phosphorylation, 250 µM, 30 min. *Nonspecific bands. (**b**) Interaction between RIPK2 and ASK1 in NOD1-HEK293A cells. After the induction of NOD1 with tetracycline, the co-transfected NOD1-HEK293A cells were processed for immunoprecipitation by anti-Flag-tag antibody beads. *mASK1* mouse ASK1, *mRIPK2* mouse RIPK2. (**c,d**) Effects of ASK1 overexpression (**c**) or ASK1 knockdown (**d**) on NOD-RIPK2 pathway activation. ASK1-overexpressing (**c**) or ASK1-knockdown (**d**) NOD1-HEK293A cells were stimulated with C12-iE-DAP (1 µg/mL) for 60 min or the indicated periods of time, respectively. Note that cycloheximide (50 µg/mL) was treated to prevent the rapid feedback synthesis of IκBα. *hASK1* human ASK1. (**e**) Schematic representation of RIPK2 deletion mutants. The numbers indicate the amino acid (a.a.) positions in wild-type (WT). Black rectangles indicate registered domains in the Pfam database (P58801). *KD* kinase domain, *IM* intermediate domain, *CARD* caspase-recruitment domain. (**f**) Binding ability of RIPK2 domain mutants to ASK1. Co-transfected HEK293A cells were processed for immunoprecipitation by anti-Flag-tag antibody beads. (**g**) Effects of ASK1 overexpression on the RIPK2-XIAP interaction. Co-transfected HEK293A cells were processed for immunoprecipitation by anti-Flag-tag antibody beads. *mXIAP* mouse XIAP. *Immunoglobulin G heavy chain. (**h**) Effects of ASK1 knockdown on K63-polyubiquitination of endogenous RIPK2 under NOD1 ligand stimulation. ASK1-knockdown NOD1-HEK293A cells were stimulated with C12-iE-DAP (1 µg/mL) for the indicated times, from which lysate K63-polyubiquitinated conjugates were purified with a tandem ubiquitin binding entity (TUBE) pull-down assay. Note that superfluous lanes were digitally eliminated from blot images in (**c,g,h**) as indicated by black lines and the uncropped images are presented in Supplementary Data.

Among the ASK1 interactor candidates, we specifically focused on RIPK2, a critical adaptor molecule in the inflammatory NOD-RIPK2 pathway because it is implicated in adipose inflammation in brown adipocytes^17^ and the interaction between ASK1 and RIPK2 in brown adipocytes suggests a potential involvement of ASK1 in brown adipose inflammation. We first selected HEK293A cells as an experimental model to investigate the functional relationship between ASK1 and the NOD-RIPK2 pathway and its molecular mechanism because HEK293A cells enable us to utilize overexpression approach with high efficiency. Upon ligand binding to NOD receptors, RIPK2 recruits its effectors, including XIAP and the TAB/TAK1 complex, and activates downstream MAPK and NF-κB signaling to induce proinflammatory responses^31^. Due to the low expression level of endogenous NOD1, stimulation with a synthetic NOD1 ligand C12-iE-DAP^32^ failed to activate JNK and p38 MAPK in the original HEK293A cells (data not shown; cf. lanes 1 and 2 in Fig. 2a). Therefore, we established a stable transfectant HEK293A cell line that expresses Myc-tagged mouse NOD1 under tetracycline treatment (NOD1-HEK293A cells). C12-iE-DAP stimulation activated the NOD-RIPK2 pathway, as indicated by enhanced phosphorylation levels of JNK and p38 MAPK in tetracycline-treated NOD1-HEK293A cells (Fig. 2a). The interaction between ASK1 and RIPK2 was confirmed in the NOD1-HEK293A cell line (Fig. 2b). Then, we investigated whether RIPK2 acts on ASK1 or vice versa. In contrast to an oxidative stress inducer H_2_O_2_, a potent ASK1 activator^33^, C12-iE-DAP did not induce ASK1 phosphorylation in NOD1-HEK293A cells (Fig. 2a), suggesting that activation of the NOD-RIPK2 pathway does not affect the kinase activity of ASK1. In contrast, overexpression of ASK1 suppressed the degradation of NF-κB inhibitor, alpha (IκBα) under C12-iE-DAP stimulation (Fig. 2c). Note that the activation of the NOD-RIPK2 pathway was monitored with the degradation of IκBα here because exogenously expressed ASK1 drastically increases the phosphorylation level of JNK and p38 MAPK^34^. Moreover, the knockdown of ASK1 enhanced C12-iE-DAP-induced IκBα degradation in NOD1-HEK293A cells (Fig. 2d). These results suggested that ASK1 suppresses the activation of the NOD-RIPK2 pathway.

To explore the inhibitory mechanism of the NOD-RIPK2 pathway by ASK1, we determined the ASK1-interacting domain of RIPK2 by coimmunoprecipitation analysis. RIPK2 is composed of three domains: the kinase domain (KD), intermediate domain (IM), and caspase recruitment domain (CARD)^35^ (Fig. 2e). Coimmunoprecipitation of wild-type ASK1 with RIPK2 domain mutants revealed that ASK1 specifically bound to KD of RIPK2 (Fig. 2f), which contains an essential residue for K63-polyubiquitination and subsequent recruitment of the TAB/TAK1 and IκB kinase (IKK) complexes^36^. Hence, we hypothesized that ASK1 inhibits the NOD-RIPK2 pathway by physically interfering with the formation of the RIPK2 complex. As an interfering effect of ASK1 on the RIPK2 complex, we evaluated the interaction between RIPK2 and one of its E3-ligases XIAP, a major contributor to K63-polyubiquitination on RIPK2 upon pathway activation^37^. The amount of XIAP that coimmunoprecipitated with RIPK2 was reduced by co-overexpression of ASK1 (Fig. 2g), suggesting that ASK1 competes with XIAP for RIPK2 interaction. By using a tandem ubiquitin binding entity (TUBE) pull-down assay^38^, we assessed the K63-polyubiquitination of RIPK2, which is induced in an early step of RIPK2 signaling complex formation upon NOD1 ligand stimulation^36^. Knockdown of ASK1 enhanced the K63-polyubiquitination of endogenous RIPK2 upon C12-iE-DAP stimulation in NOD1-HEK293A cells (Fig. 2h). These data collectively suggest that ASK1 downregulates the NOD-RIPK2 pathway at least in part by impeding RIPK2 from forming a functional signaling complex.

### ASK1 suppresses the NOD-RIPK2 pathway and cytokine induction in brown adipocytes

**Figure 3:**
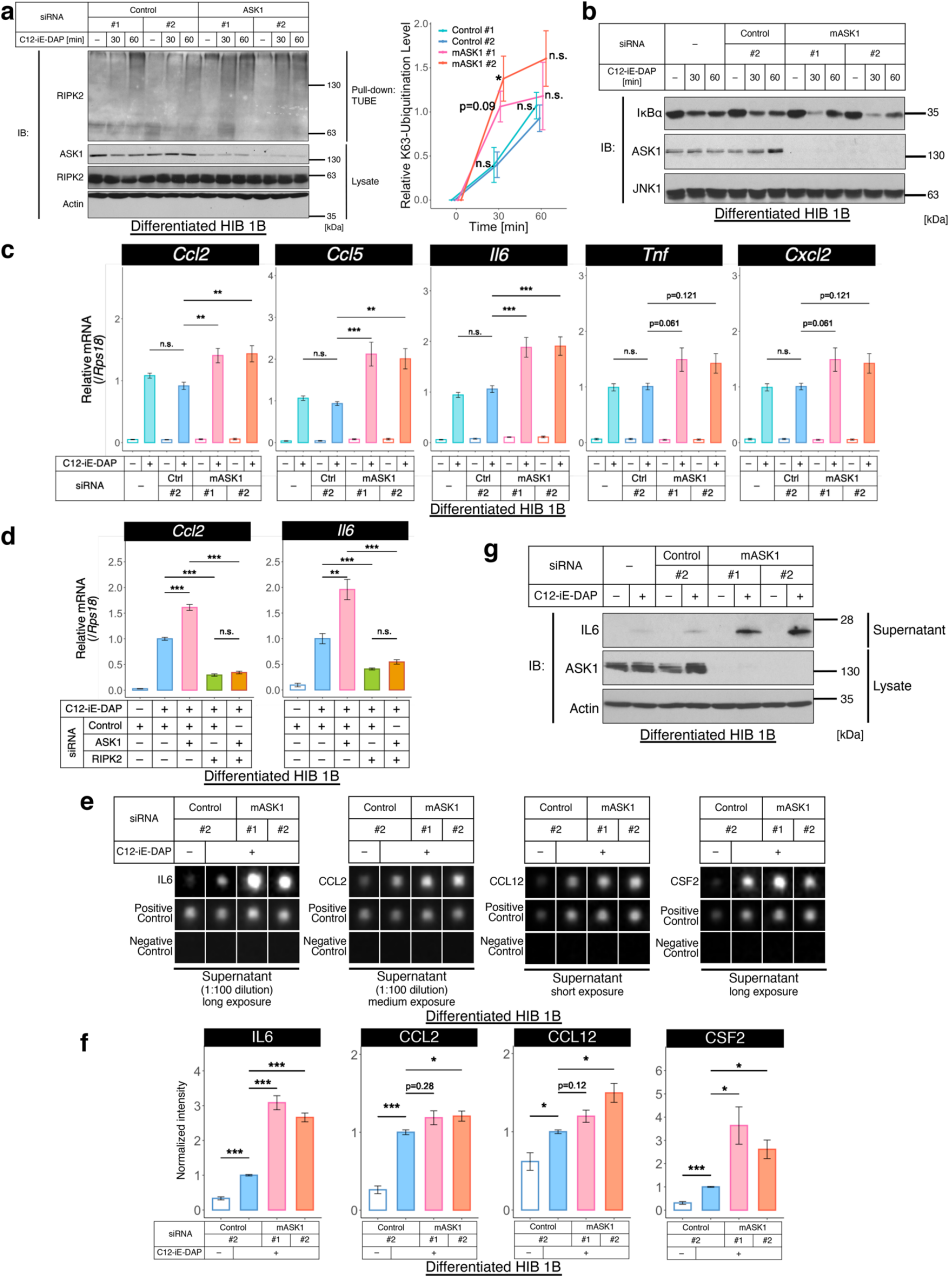
ASK1 suppresses the NOD-RIPK2 pathway and cytokine induction in brown adipocytes. (**a,b**) Effects of ASK1 knockdown on the K63-polyubiquitination of RIPK2 (**a**) or the activation of the NOD-RIPK2 pathway (**b**) in brown adipocytes. ASK1-knockdown HIB 1B cells were treated with C12-iE-DAP (10 µg/mL) for the indicated times. The right graph in (**a**) indicates the quantification of western blot, and statistical analysis was performed against Control #2 at each time point. *JNK* loading control in (**b**). (**c,d**) Effects of ASK1 knockdown (**c**) or ASK1/RIPK2 double knockdown (**d**) on the relative mRNA levels of inflammatory cytokines under NOD-RIPK2 pathway activation. Differentiated HIB 1B cells were treated with C12-iE-DAP (10 µg/mL, 6 h). (**e–g**) Effects of ASK1 knockdown on cytokine secretion from NOD-RIPK2 pathway-activated cells. Supernatant (**e**), or supernatant and cell lysates (**g**) from C12-iE-DAP (10 µg/mL, 8 h)-treated HIB 1B cells were subjected to cytokine antibody array (**e**) or immunoblotting (**g**), respectively. The images of cytokine antibody array were quantified in (**f**). See Supplementary Fig. S1 for the other cytokines. Data are represented as the mean ± SEM. *n* = 4 in (**a**), *n* = 9 (pooled from 3 independent experiments) in (**c**), *n* = 8 (pooled from 4 independent experiments) in (**d**), *n* = 6 (pooled from 3 independent experiments) for IL6, CCL2 and *n* = 8 (pooled from 4 independent experiments) for CCL12, CSF2 in (**f**). *P < 0.05, **P < 0.01, ***P < 0.001, *n.s.* not significant, according to two-tailed Dunnett’s test (**a,c**) or two-tailed Welch’s *t*-test with the Bonferroni correction (**d,f**). *Ctrl* control.

Since the interaction between ASK1 and RIPK2 was originally identified in brown adipocytes, we next investigated the role of ASK1 in the NOD-RIPK2 pathway with an experimental model of brown adipocytes, HIB 1B cells^29^. As similar with in NOD1-HEK293A cells, ASK1 knockdown in differentiated HIB 1B cells enhanced the K63-polyubiquitination of RIPK2 under NOD1 ligand stimulation (Fig. 3a). Moreover, knockdown of ASK1 in brown adipocytes augmented the C12-iE-DAP-induced degradation of IκBα (Fig. 3b). Note that the maximum effects of ASK1 on the NOD-RIPK2 pathway were observed in different time point between NOD1-HEK293A cells and HIB 1B cells (Fig. 2d,h vs. Fig. 3a,b), implying that the involvement of ASK1 in the NOD-RIPK2 pathway is conserved across cell lines but the NOD-RIPK2 pathway is finetuned differently (e.g., feedback mechanism). We further examined whether the suppressive effects of ASK1 on the NOD-RIPK2 pathway are physiologically relevant to inflammatory cytokine production in brown adipocytes by measuring the mRNA levels of chemokines^17,39^. ASK1 knockdown exhibited the significant upregulation for the induction of chemokine (C-C motif) ligand 2 (*Ccl2*, also known as MCP-1), *Ccl5* (also known as RANTES), and interleukin 6 (*Il6*) mRNAs, and the upregulating tendency for tumor necrosis factor (*Tnf*, typically known as TNFα) and chemokine (C-X-C motif) ligand 2 (*Cxcl2*) in C12-iE-DAP-treated HIB 1B cells (Fig. 3c). This upregulation of inflammatory cytokines observed under single knockdown of ASK1 was abolished when both ASK1 and RIPK2 were knocked down (Fig. 3d). These results suggest that ASK1 suppresses cytokine production in a NOD-RIPK2-dependent manner.

Subsequently, we investigated the suppressive effect of ASK1 on cytokine production in brown adipocytes at the protein level using a cytokine antibody array targeting 22 mouse cytokines. The systematic approach revealed that stimulation of HIB 1B cells with C12-iE-DAP significantly upregulated IL6, CCL2, CCL5, CCL12, colony stimulating factor 2 (CSF2, also known as GM-CSF), CSF3 (also known as GCSF), IL9, IL12 p40/p70, IL17 and thrombopoietin (also known as THPO) while significantly downregulated soluble TNF receptor-1 (sTNFR1) and vascular endothelial growth factor (VEGF) (Fig. 3e,f, Supplementary Fig. S1), some of which were consistent with the previous report^17^. Among them, ASK1 knockdown showed significant increase in the C12-iE-DAP-dependent upregulation of IL6 and CSF2 and increasing tendency in that of CCL2 and CCL12 (Fig. 3e,f). Of note, CCL5 was not significantly increased by ASK1 knockdown at the protein level (Supplementary Fig. S1) in contrast to the mRNA result (Fig. 3c), but we cannot rule out the possibility that the effect of ASK1 on CCL5 accumulation can be detected only at later time points due to protein turnover time course of CCL5. Interestingly, the C12-iE-DAP-dependent upregulation of thrombopoietin was significantly decreased by ASK1 knockdown (Supplementary Fig. S1), possibly implying that the effect of ASK1 knockdown on thrombopoietin may be derived via direct ASK1 activity for basal p38/JNK MAPK pathway although we need more detailed analyses. As a representative validation of the cytokine antibody array assay, we confirmed the increased secretion of IL6 by ASK1 knockdown using western blot (Fig. 3g). Altogether, these results suggest that ASK1 suppresses the release of not global but some specific cytokines in brown adipocytes.

### ASK1 does not suppress the NOD-RIPK2 pathway and cytokine production in white adipocytes

Mammalian adipocytes are classified into two classes: white adipocytes and brown adipocytes^4,5^. Our finding that ASK1 suppresses the NOD-RIPK2 pathway in brown adipocytes led us to investigate the involvement of ASK1 in the NOD-RIPK2 pathway in white adipocytes; i.e., we differentiated 3T3-L1 cells^13^ as an experimental model of white adipocytes and examined the effect of ASK1 knockdown on the NOD-RIPK2 pathway. Stimulation of the differentiated 3T3-L1 cells with C12-iE-DAP activates the NOD-RIPK2 pathway, as indicated by degradation of IκBα (Supplementary Fig. S2a). However, against our expectation, the knockdown of ASK1 did not show the significant enhancement of IκBα degradation (Supplementary Fig. S2a). In addition, while stimulation of 3T3-L1 cells with C12-iE-DAP induced the expression of pro-inflammatory cytokines *Ccl2*, *Ccl5* and *Il6* as previously reported^13^, ASK1 knockdown did not enhance inflammatory cytokine induction under ligand stimulation (Supplementary Fig. S2b). These results indicate that ASK1 regulates the NOD-RIPK2 pathway in a cell type-dependent manner.

### Inhibition of the NOD-RIPK2 pathway contributes to maintenance of thermogenic potential in brown adipocytes

Cell type-specific ASK1 suppression implies some physiological meaning of the NOD-RIPK2 pathway in brown adipocytes. Several studies have reported that acute or chronic activation of pattern recognition receptors, namely, Toll-like receptor 2 (TLR2), TLR4 and NOD1, attenuate the expression of brown adipocyte markers in brown adipocytes^17,40^. Hence, we hypothesized that suppression of the NOD-RIPK2 pathway by ASK1 may contribute to the thermogenic function in brown adipocytes. However, because the PKA-ASK1-p38 axis is involved in the maturation of brown adipocytes^19^, it may not be easy to distinguish the roles of ASK1 in the NOD-RIPK2- and PKA-p38-dependent regulations of brown adipocytes using a simple knockdown experiment of ASK1 in brown adipocytes. Adipose inflammation is aggravated by local cross-talk between adipocytes and infiltrated macrophages^41^, and proinflammatory cytokines secreted from macrophages are involved in paracrine regulation of thermogenic function in brown adipocytes^42,43^. These cytokines can also be secreted from brown adipocytes^44^. Therefore, we instead established an experimental model to explore a role of ASK1 in the cytokines-mediated regulation of thermogenic potential by measuring β_3_-adrenergic receptor responsiveness (Supplementary Fig. S3a). Briefly, the inflammatory cytokine-containing culture medium (conditioned medium) was collected from brown adipocytes that were treated with the NOD-RIPK2 pathway activator and/or siRNA against ASK1 in advance (“donor cells”) (cf. Fig. 3e–g). Subsequently, another set of brown adipocytes (“acceptor cells”) was stimulated with a β_3_-adrenergic receptor agonist following exposure to the conditioned medium, and the induction of brown adipocyte markers in acceptor cells was evaluated. We first examined whether our experimental system could model the inflammatory environments where thermogenic markers are downregulated in brown adipocytes. Compared with control media, conditioned medium from the C12-iE-DAP-treated donor cells significantly suppressed the brown adipocyte markers *Ucp1*, *Prdm16* and *Cox4i1* induced by CL316,243, a β_3_-adrenergic receptor-specific agonist^45^, in the acceptor HIB 1B cells (Supplementary Fig. S3b), suggesting that our model enables us to evaluate the inflammatory BAT-derived intercellular effects on the thermogenic function of BAT. Thus, we determined the effect of ASK1 knockdown in donor HIB 1B cells on the responsiveness to the β_3_-adrenergic receptor agonist in acceptor cells. ASK1 knockdown in donor HIB 1B cells aggravated the inhibitory effect of C12-iE-DAP-treated conditioned medium on brown adipocyte markers upon CL316,243 administration in acceptor HIB 1B cells (Supplementary Fig. S3c). Altogether, our results support the hypothesis that the inhibitory effect of ASK1 on the NOD-RIPK2 pathway is involved in maintaining the thermogenic potential of brown adipocytes in an inflammatory environment.

## Discussion

In this study, we established a novel chemical pull-down MS method and identified RIPK2 as an ASK1 interactor in brown adipocytes. The affinity purification-MS (AP-MS) method has been one of the representative footholds to characterize the regulations and functions of a protein of interest, and we have indeed conducted the AP-MS analyses using samples of tagged-ASK1-overexpressing HEK293A cells^27,46^. However, none of the previous trials identified RIPK2 as an ASK1 interactor. Although purification of overexpressed protein is most commonly used in AP-MS, the method often faces several issues. For instance, tagging at the terminus of a protein may affect the conformation or subcellular localization of the protein and impede the access of its binding partners^47^, which reduces the protein interactions in cells and also in solution through pull-down step. Overexpressed proteins can also interact with artificial partners in cells, which makes it difficult to distinguish genuine endogenous interactors. In addition, a strong affinity between avidin and biotin (*K*_D_ ~ 10^−15^ [M]), one of the most commonly used combinations for chemical pull-down systems, makes it difficult to elute the protein complex without the alteration of pH or temperature or the addition of denaturants^48^, which is not optimal for elution condition. Besides, purification of endogenous protein complexes depends largely on the availability of antibodies for pull-down assays; thus, there have been only a few reports on identifying components of endogenous signalosomes. We propose that our novel ASKA pull-down MS method overcomes major drawbacks in the typical AP-MS methods and hence is a powerful AP-MS option that is applicable to a broad range of endogenous kinases when identifying genuine components of its signalosome. To utilize the high specificity of 1NA-PP1 to the as-kinase, ASKA technology introduces mutations in the ATP-binding pockets^22,49^. The structure and sequence of the ATP-binding pocket are so highly conserved that this kinase modification methodology has been applied to numerous kinases. The recent advancement in genome editing technology (e.g., CRISPR-Cas9 system^50^) provides an easier environment to introduce gene modification. Previous reports proved that ASKA modification did not considerably affect the ATP-binding ability or kinase activity of the as-kinase^23,51^, and we also demonstrated that purification of as-kinase with 1NA-PP1 did not significantly alter the total size of the kinase signalosome (Fig. 1g). We believe the ASKA pull-down MS method will facilitate protein–protein interaction analyses of any desired kinase, and is highly promising as a tool to drive kinase research.

BAT is a unique organ for energy expenditure through thermogenesis. Inflammation in brown adipocytes is of great research interest but remains relatively unknown compared to white adipose inflammation. In this study, we showed that ASK1 suppresses the inflammatory NOD-RIPK2 pathway and cytokine secretion in brown adipocytes. While ASK1 has been reported as a critical regulator of inflammation^23,34,52^, most of these reports demonstrated that ASK1 promotes the inflammatory response. Our finding is unique in that ASK1 suppresses the inflammatory response. A remaining question lies in the detailed regulatory mechanism of the NOD-RIPK2 pathway by ASK1. The simplest explanation would be that ASK1 physically hinders the modification of RIPK2 or recruitment of its downstream effector molecules. We showed that ASK1 specifically bound to the kinase domain of RIPK2 (Fig. 2f) and inhibited the recruitment of an E3 ubiquitin ligase XIAP and the K63-polyubiquitination of RIPK2 (Fig. 2g,h). The kinase domain of RIPK2 (18–290 amino acids; Fig. 2e) harbors several important residues involved in downstream activation. For instance, S176 in the activation loop is autophosphorylated under NOD ligand stimulation^53^. K63-type polyubiquitination on K209 is important for the recruitment of the TAB/TAK1 complex and the subsequent activation of downstream NF-κB signaling^36^. The binding interface between XIAP and RIPK2 was mapped in the kinase domain of RIPK2^54^. Hence, binding of ASK1 to the kinase domain might function as a direct physical obstruction or induce structural change of RIPK2 to block these modifications.

**Figure 4:**
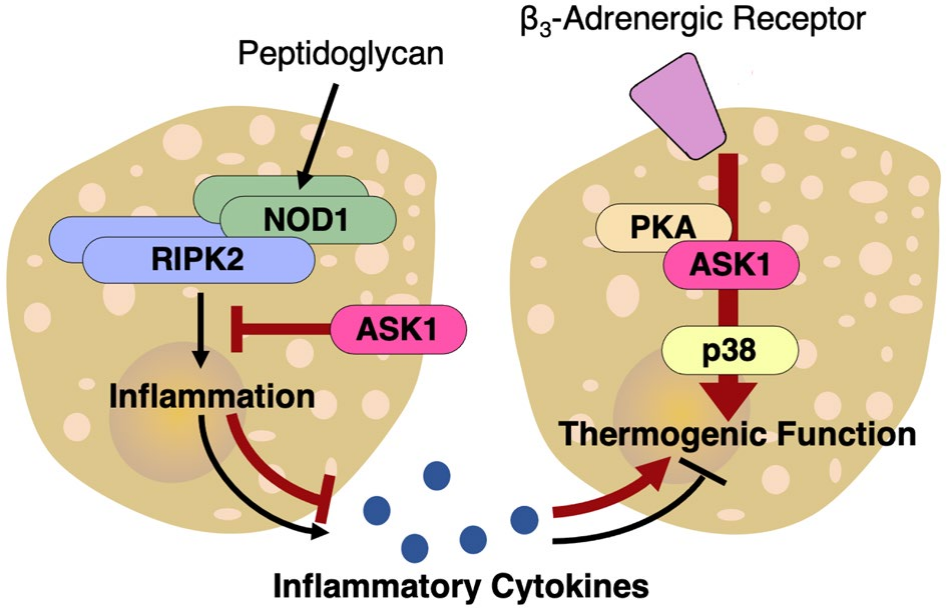
Hypothetical model. Through interacting with RIPK2, ASK1 negatively regulates the NOD-RIPK2 pathway and inflammatory cytokine production in brown adipocytes. Along with the maturation-enhancing effect of ASK1 via the PKA-ASK1-p38 axis under β_3_-adrenergic receptor stimulation^19^, this regulation would contribute to maintaining brown adipocyte function under inflammation.

As a potential biological significance of the ASK1-dependent RIPK2 regulation in BAT, our in vitro model with HIB 1B cells suggested that the suppression of inflammation in brown adipocytes by ASK1 contributes to the intercellular maintenance of thermogenic capacity in brown adipocytes (Supplementary Fig. S3). The result is in accordance with our previous report that ASK1 is involved in brown adipocyte maturation via activation of the PKA-ASK1-p38 axis^19^; that is, our studies underpin the multifaceted regulatory mechanism of brown adipocyte maintenance by ASK1 (Fig. 4). It is reasonable that a single signaling molecule controls the same physiological function under different situations—in this case, thermogenic gene expression under normal differentiation processes and under inflammation. However, we should still keep the limitations of our in vitro model in mind. For instance, although adipose inflammation is aggravated by local cross-talk between adipocytes and immune cells in the whole body, our in vitro model does not take the effects of immune cells into account. Moreover, we tested our hypothesis using HIB 1B cells. Although utilized as a brown adipocyte cell line, HIB 1B cells express limited amount of brown adipocyte markers such as Ucp1 and β_3_-adrenergic receptor compared to primary brown adipocytes derived from rodents^29^. Further studies with primary culture cells and in vivo systems will be needed to validate our hypothesis and test the biological effect size in vivo.

As another clue for the biological significance of our findings, we found that ASK1 does not suppress the NOD-RIPK2 pathway in white adipocytes (Supplementary Fig. S2). Activation of the NOD-RIPK2 pathway in white adipocytes induces insulin resistance, that is, it seems to be maladaptive for energy homeostasis^13,15^. However, inflammation in white adipocytes can also have a beneficial effect on promoting WAT expansion and remodeling, which limits the permeability of bacterial fragments as an intestinal barrier^55^. Hence, adaptive reprogramming in WAT against increased energy uptake^55^ may be blocked by inhibition of the NOD-RIPK2 signaling in white adipocytes. On the other hand, inflammation in BAT suppresses UCP1 expression in brown adipocytes and thus limits the energy expenditure under inflammation^17^. BAT-specific regulation of the NOD-RIPK2 pathway by ASK1 may contribute to effectively maintaining the thermogenic function of brown adipocytes without impairing the inflammation-driven reprogramming of WAT. The molecular mechanism of how ASK1 achieves brown adipocyte-specific regulation of the NOD-RIPK2 pathway still needs future investigation.

From a macro perspective, adipose inflammation is a crucial hub for obesity and metabolic dysregulation. Chronic low-grade inflammation of adipose tissue, characterized by increased secretion of inflammatory cytokines and infiltration of macrophages and other types of immune cells, is observed under obesity and regarded as a trigger of metabolic disorders, including type 2 diabetes and cardiovascular diseases^3^. The physiological ligand of NODs, peptidoglycan, is considered to be derived from gut microbiota and translocated from the luminal side of the mucosa into the host circulation^11^. High-fat diet feeding affects gut microbiota and enhances intestinal permeability^12,56^. Besides, *Nod1* and *Nod2* double knockout mice are protected from high fat diet-induced insulin intolerance^15^, and several reports pointed out that the NOD-RIPK2 pathway is activated in adipose tissue from patients with metabolic syndrome or diabetes^57,58^. These lines of evidence imply that obesity may facilitate inflammation and metabolic dysregulation in a NOD-RIPK2-dependent manner. In this study, we showed that ASK1 downregulated the NOD-RIPK2 pathway (Fig. 3, Supplementary Fig. S3), which suggests the potential function of ASK1 as an adipose inflammation suppressor by regulating the NOD-RIPK2 pathway. At the same time, ASK1 expression in adipose tissue is upregulated under obesity both in human^59,60^ and in mice^61^, and kinase activity of ASK1 is upregulated in mice under high fat diet-feeding^62^, which rather propose that ASK1 expression and/or activation promote adipose inflammation. Alternatively, our results may point out the existence of latent beneficial aspect of ASK1 upregulation through tuning the NOD-RIPK2 pathway under obesity. As a potential relevant finding to this assumption, we previously demonstrated that global *Ask1* knockout mice showed impaired glucose clearance compared to wild-type mice under high-fat diet treatment, and the phenotype was more robust in severely obese animals^20^. Unexpectedly, however, adipocyte-specific *Ask1* knockout mice exhibited comparable glucose clearance with wild-type^20^, which denies the potential influence of ASK1 on blood glucose level under obesity through downregulating the pro-inflammatory NOD-RIPK2 pathway in adipocytes. Nevertheless, considering the confounding example of body weight in the phenotype of *Ask1* knockout mice and complexity of obesity-induced inflammatory response in vivo, we should keep in mind that negative regulation of the NOD-RIPK2 pathway by ASK1 in brown adipocytes may contribute to unidentified whole-body phenotype in some obesity-associated situation.

In conclusion, our work demonstrated an example application of the ASKA pull-down MS method and revealed a novel regulatory mechanism in adipose inflammation that ASK1 suppresses inflammatory NOD-RIPK2 signaling in brown adipocytes. As a crucial hub of adipose inflammation and thermogenic gene induction, the NOD-RIPK2 pathway in brown adipocytes would be an attractive therapeutic target against obesity-associated diseases. Further investigation of the regulatory mechanism of the NOD-RIPK2 pathway by ASK1 may provide new insight into the clinical approach to obesity.

## Methods

### Antibodies and reagents

Antibodies used in this study are listed in Supplementary Table S1^63^.

C12-iE-DAP (#tlrl-c12dap) was purchased from InvivoGen, dissolved at 10 mg/mL in dimethyl sulfoxide (DMSO; Sigma-Aldrich, #D5879) and dissolved at a final concentration of 1000× in LAL water (InvivoGen, #h2olal-1.5). CL316,243 (#sc-203895) was purchased from Santa Cruz Biotechnology, and H_2_O_2_ (#081-04215) was purchased from Wako Pure Chemical Industries and dissolved at a final concentration of 1000× in ultrapure water. Cycloheximide was purchased from Sigma-Aldrich and dissolved at a final concentration of 100 mg/mL in DMSO.

### Cell lines and cell culture

HEK293A cells (Invitrogen) were cultured in DMEM-high glucose (Sigma-Aldrich, #D5796) supplemented with 10% fetal bovine serum (FBS, biowest). HIB 1B cells, a kind gift from Dr. Bruce M. Spiegelman (Harvard Medical School and Dana-Farber Cancer Institute), were cultured in DMEM-high glucose supplemented with 10% FBS. Of note, to eliminate mycoplasma, the original HIB 1B cells were treated with MynoxGold (Minerva Biolabs, #10-0201) and subcloned by limiting dilution. 3T3-L1 cells, a kind gift from Dr. Shin-Ichiro Takahashi (The University of Tokyo), were cultured in DMEM-high glucose supplemented with 10% calf serum (Sigma-Aldrich, #C8056) and 100 units/mL penicillin G (Meiji Seika, #6111400D2039). Tetracycline-inducible mNOD1-6Myc-stably expressing HEK293A (NOD1-HEK293A) cells were established as previously described^64^ and cultured in DMEM-high glucose supplemented with 10% FBS, 2.5 µg/mL blasticidin (Invitrogen, #A1113903), and 50 µg/mL Zeocin (Invitrogen, #R25001). To induce mNOD1-6Myc, cells were pretreated with 1 μg/mL tetracycline (Sigma-Aldrich, #T7660) 1 day before assays.

For brown adipocyte differentiation, HIB 1B cells were plated at 1.0 × 10^5^ cells/mL on day 4. Cells were exposed to differentiation induction medium (DMEM-high glucose containing 1 nM triiodo-l-thyronine (T3; Sigma-Aldrich, #T2877), 20 nM insulin (Sigma-Aldrich, #I1882), 5 µM dexamethasone (Sigma-Aldrich, #D4902), 0.125 mM indomethacin, 0.5 mM 3-isobutyl-1-methylxanthine (IBMX; Sigma-Aldrich, #I5879), 1 µM rosiglitazone (Sigma-Aldrich, #R2408), 20 mM HEPES–NaOH (pH 7.4), 20% FBS) on day 0. The medium was changed to differentiation enhancement medium (DMEM-high glucose containing 1 nM T3, 20 nM insulin, 20 mM HEPES–NaOH (pH 7.4), 20% FBS) on days 2, 4 and 6. The medium was replaced with fresh DMEM-high glucose supplemented with 20% FBS 1 h prior to starting experiments.

For white adipocyte differentiation, 3T3-L1 cells were plated at 1.0 × 10^5^ cells/mL on day 4. Cells were exposed to differentiation induction medium (DMEM-high glucose containing 1.7 mM insulin, 250 nM dexamethasone, 0.5 mM IBMX, 10% FBS) on day 0. The medium was changed to differentiation enhancement medium (DMEM-high glucose containing 1.7 mM insulin, 10% FBS, DE) on days 2, 4, and 6, resuspended to 5.0 × 10^5^ cells/mL on day 8 with DE, and replaced with fresh DE on day 10. The medium was replaced with fresh DMEM-high glucose supplemented with 10% FBS 1 h prior to starting the experiments.

Primary brown fat stromal vascular fraction (SVF) was isolated from newborn *Ask1*^ASKA^ knock-in mice and differentiated into brown adipocytes as previously reported^19^. All experiments were performed in accordance with protocols approved by the Animal Research Committee of the Graduate School of Pharmaceutical Sciences, The University of Tokyo (Tokyo, Japan).

### In silico analysis of the ASK1 ATP-binding pocket

The previously reported crystal structure of the ASK1 kinase domain (PDB: 2CLQ)^24^ was analyzed using AutoDock 4.0 (Scripps Research) software to calculate the depth of the ATP-binding pocket.

### Synthesis of 1NA-PP1 derivatives

1NA-PP1-L1 and 1NA-PP1-L2 were synthesized as described in Supporting Information (Supplementary Note)^65,66^.

### Mass spectrometry analysis

The as-ASK1 complex was purified from primary brown adipocytes (day 4). After concentration by TCA precipitation, the purified as-ASK1 samples were dissolved in guanidine hydrochloride and digested with lysyl endopeptidase (Lys-C; Wako Chemicals USA) and then further digested with trypsin (Sigma-Aldrich). All samples were analyzed by a direct nanoflow liquid chromatography (LC) system coupled to a time-of-flight mass spectrometer (Triple TOF 5600+; AB Sciex) as previously described^67^. We regarded the identified protein as an interactor candidate of ASK1: (1) if the protein was uniquely mapped from the obtained peptides and (2) if the number of peptides obtained from 1NA-PP1-eluted samples was larger than that from DMSO-eluted negative control samples.

In a meta-analysis of different pull-down MS results, the identified mouse proteins in the ASKA pull-down MS method were converted to human orthologs using the NCBI Entrez database in a highly conservative manner; two proteins, Pcdhgb8 and Vmn1r87, were unable to be assigned to human orthologs. For the Flag-tag pull-down MS result^27^, we set criteria for interactor candidates of ASK1: (1) if the protein was uniquely mapped from the obtained peptides and (2) if the number of peptides obtained from the wild-type ASK1-transfected samples was larger than that from mock negative control samples. Regarding the other Flag-tag pull-down MS result^28^, we regarded the listed protein as an interactor candidate of ASK1 if the significance analysis of interactome (SAINT) score was more than 0.6.

### Expression plasmids

Expression plasmids for this study were constructed by standard molecular biology techniques, and all constructs were verified by sequencing. A mouse ASK1 cDNA (CDS of NM_008580.4 harboring four silent mutants in its nucleotide sequence and K203R mutant and lacking stop codon) was previously cloned and subcloned into pcDNA3 (Invitrogen) with a C-terminal HAsl tag^46^. cDNAs of mouse RIPK2 (CDS of NM_138962.4), mouse NOD1 (CDS of NM_172729.3), and mouse XIAP (CDS of NM_001301639.1) were subcloned into pcDNA3/GW or pcDNA4 (Invitrogen) with an N- or C-terminal Flag, a C-terminal 6Myc, or an N-terminal YFP. RIPK2 mutants KD (CDS of NM_138962.4 with c.874_1,617del), IM (CDS of NM_138962.4 with c.1_873del, c.1291_1617del), and CARD (CDS of NM_138962.4 with c.1_1302del) were constructed from full-length RIPK2 and subcloned into pcDNA3/GW with an N-terminal Flag and C-terminal YFP.

### Transfections

Plasmid transfections were performed with polyethylenimine “MAX” (Polysciences, #24,765) when HEK293A cells were grown to 80–95% confluency, according to a previously described protocol^68^ with minor optimization. In certain experiments, after 4–6 h of transfection, the cells were cultured in fresh medium for another 40 h to reduce cytotoxicity.

siRNA transfections were carried out by reverse transfection using Lipofectamine RNAiMAX (Invitrogen, #133778-150) according to the manufacturer’s instructions. For siRNA transfection of NOD1-HEK293A cells, cells were reverse transfected with 10 nM siRNA on the day of seeding. For siRNA transfection of HIB 1B cells, cells were resuspended in DE buffer and transfected with 16.7 nM siRNAs on day 4 or resuspended in DI buffer on day 0 and in DE buffer on day 4 with reverse transfection of 16.7 nM siRNAs. For siRNA transfection of 3T3-L1 cells, cells were resuspended in DE buffer and transfected with 4.17 nM siRNAs on day 8. siRNA sequences are listed in Supplementary Table S2.

### Cell lysis and immunoblotting

Cells were lysed in lysis buffer (20 mM Tris–HCl pH 7.5, 150 mM NaCl, 10 mM EDTA, 1% sodium deoxycholate, and 1% Triton X-100) supplemented with 1 mM phenylmethylsulfonyl fluoride (PMSF), 5 μg/mL leupeptin, and phosphatase inhibitor cocktail (20 mM NaF, 30 mM β-glycerophosphatase, 2.5 mM Na_3_VO_4_, 3 mM Na_2_MoO_4_, 12.5 μM cantharidin and 5 mM imidazole). For the samples in Fig. 1I, cells were lysed in lysis buffer containing 10 mM *N*-ethylmaleimide (Tokyo Chemical Industry, #E0136) dissolved in ethanol (Wako Pure Chemicals Industries, #057000451). Cell extracts were clarified by centrifugation, and supernatants were sampled by adding SDS sample buffer. In certain experiments, 5% volume/volume β-mercaptoethanol (Nacalai Tesque, #21438082) was added to SDS sample buffer.

For the supernatant in Fig. 3g, culture medium was substituted into medium supplemented with 0.2% FBS prior to experiments. Culture medium was collected and centrifuged for 5 min to separate the supernatant from a pellet. The supernatants were mixed with fourfold volumes of ice-cold acetone (Wako Pure Chemicals Industries, #012-00343), followed by incubation at − 20 °C over 2 h and subsequent sedimentation through centrifugation. The samples were dissolved in SDS sample buffer.

After boiling at 98 °C for 3 min, the samples were resolved by SDS-PAGE and electroblotted onto a BioTrace PVDF membrane (Pall) or Immobilon-P membrane (Millipore, #IPVH00010). The membranes were blocked with 2% skim milk (Megmilk Snow Brand) in TBS-T (20 mM Tris–HCl pH 8.0, 137 mM NaCl and 0.1% Tween 20) and probed with the appropriate primary antibodies diluted by 1st antibody-dilution buffer (TBS-T supplemented with 5% BSA (Iwai Chemicals, #A001) and 0.1% NaN_3_ (Nacalai Tesque, #312-33)). After replacing and probing the appropriate secondary antibodies diluted with skim milk in TBS-T, antibody-antigen complexes were detected on X-ray films (FUJIFILM, #47410-07523, #47410-26615 or #47410-07595) using an ECL system (GE Healthcare). Quantification was performed against the raw digital images with densitometry using Fiji/ImageJ software^69^.

### Cytokine antibody array

Cytokines secreted into culture medium were quantified using Mouse Cytokine Antibody Array (Membrane, 22 targets) (abcam, #ab133993) following the manufacturer’s protocol. Briefly, culture medium was substituted into fresh medium supplemented with 0.2% FBS prior to experiments, and culture medium was collected 8 h after the C12-iE-DAP stimulation (10 µg/mL). 100-fold diluted culture medium was incubated with an anti-cytokine antibody-arrayed membrane overnight at 4 °C. The target cytokines-trapped membrane was further incubated with the biotin-conjugated anti-cytokine antibody overnight at 4 °C, followed by the reaction with horseradish peroxidase (HRP)-streptavidin. The HRP-labelled cytokines was detected with chemiluminescence using a chemiluminescence imaging system FUSION SOLO.7S.EDGE (Vilber). Because the detection range in this kit is dependent on each cytokine, the detected membrane was adequately washed with TBS-T and iterated the above detection procedure for the undiluted culture medium.

The obtained images were adjusted by rolling ball background subtraction and quantified with using ImageJ/Fiji software. The quantified values were scaled with the values of negative controls and positive controls in each membrane.

### Statistical analysis

The data are summarized as the mean ± SEM. No statistical method was utilized to predetermine the sample size. Statistical tests, the number of samples and the sample sizes are indicated in each figure legend. Statistical tests were performed using R with RStudio software, and *P* < 0.05 was considered statistically significant. The investigators were not blinded to allocation during experiments and outcome assessments. The experiments were not randomized. However, the experiments were performed across different passages of cells, and the cells in the control and treated groups were seeded from the same population of cells.

Other methods in this work, including surface plasmon resonance assay, gel filtration column chromatography, preparation of 1NA-PP1-Lx-immobilized beads, purification of the as-ASK1 signalosome, quantitative RT-PCR analysis, coimmunoprecipitation assay and TUBE pull-down assay, are described in Supplementary Methods. All methods are reported in accordance with the ARRIVE guidelines (https://arriveguidelines.org), but further information and requests for resources and reagents should be directed to K.W. and H.I.

## Supplementary Information

Supplementary Information 1.

Supplementary Information 2.

## Data Availability

The authors declare that all data supporting the findings of this study are available within the main article or the supplementary materials. Source data are provided with this paper as Supplementary Data.
