# Supplementary material for: ASKA technology-based pull-down method reveals a suppressive effect of ASK1 on the inflammatory NOD-RIPK2 pathway in brown adipocytes﻿
Source: Sci Rep. 2021 Nov 10;11:22009. doi: 10.1038/s41598-021-01123-7 (PMC8581049; doi:10.1038/s41598-021-01123-7)
Supplement: Supplementary file 2 — Supplementary Information 2. [file 41598_2021_1123_MOESM2_ESM.pdf]

Supplementary Information

ASKA technology-based pull-down method reveals a suppressive effect of ASK1 on the inflammatory NOD-RIPK2 pathway in brown adipocytes

Saki Takayanagi, Kengo Watanabe, Takeshi Maruyama, Motoyuki Ogawa, Kazuhiro Morishita, Mayumi Soga, Tomohisa Hatta, Tohru Natsume, Tomoya Hirano, Hiroyuki Kagechika, Kazuki Hattori, Isao Naguro, and Hidenori Ichijo

This PDF file includes:

Supplementary Note
Supplementary Methods
Supplementary Figures S1 to S3
Supplementary Tables S1 to S3

Other Supplementary Information file in this study includes:

Supplementary Data

Supplementary Note

Synthesis of 1NA-PP1 derivatives

General

All reagents were purchased from Sigma-Aldrich Chemical, Tokyo Kasei Kogyo, Wako Pure Chemical Industries, and Kanto Kagaku. Silica gel for column chromatography was purchased from Kanto Kagaku. NMR spectra were recorded on Bruker AVANCE 400 or Bruker Advance 500 spectrometer. Mass spectral data was obtained on Brucker Daltonics microTOF-2focus in the positive ion detection modes.

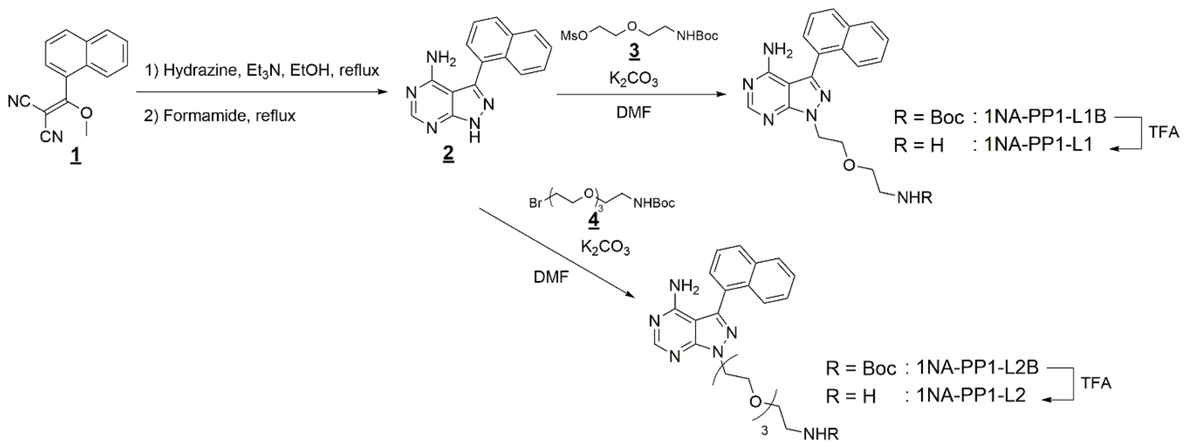

Scheme S1. Synthesis of 1NA-PP1 derivatives

Synthesis of 2

Hydrazine hydrochloride (0.19 g, 1.9 mmol) and trimethylamine (0.78 mL, 5.6 mmol) was added to a solution of **1**[59] (0.43 g, 1.9 mmol) in ethanol (15 mL). After refluxing for 3 h, the solvent was evaporated and water was added. The mixture was extracted with ethyl acetate. The organic phase was washed with brine, dried over sodium sulfate, and evaporated to dryness. The residue was suspended in formamide (10 mL), and the reflux for 14 h under argon atmosphere. After cooled to room temperature, the reaction mixture was poured into water and the precipitate was collected as compound **2** (0.40 g, 83%). This compound was utilized in next reaction without further purification.

^{1}H NMR (400 MHz, DMSO-d$_6$) δ 13.97 (br, 1 H), 8.24 (s, 1 H), 8.08-8.03 (m, 2 H), 7.83 (d, J = 8.4, 1H), 7.67-7.51 (m, 4 H); HRMS (ESI) calcd for $C_{15}H_{12}N_5$ $(M+H)^+$ 262.1087. Found 262.1082.

Synthesis of 1NA-PP1-L1B

2 (0.10 g, 0.39 mmol), **3**[60] (0.11 g, 0.38 mmol) and potassium carbonate (0.21 g, 1.5 mmol) were suspended in DMF (8 mL), and the reaction mixture was stirred at 60 ºC for 4.5 h. The mixture was poured into water, and extracted with ethyl acetate. The organic phase was wash with water and brine, dried over sodium sulfate and evaporated to dryness. Purification of the residue by silica gel chromatography (CH_2Cl_2 /methanol = 19/1) gave 1NA-PP1-L1B (56 mg, 33%).

^{1}H NMR (400 MHz, $CDCl_3$) δ 8.48 (s, 1 H), 8.00-7.91 (m, 3 H), 7.69-7.50 (m, 4 H), 5.41 (br, 1 H), 5.02 (br, 2 H), 4.70 (t, J = 5.6, 2 H), 4.02 (t, J = 5.6, 2 H), 3.59 (t, J = 5.2, 2 H), 3.31-3.27 (m, 2 H), 1.45 (s, 9 H); ^{13}C NMR (125 MHz, $CDCl_3$): δ 157.6, 156.5, 156.1, 154.7, 142.8, 133.9, 131.5, 130.9, 129.9, 128.6, 128.4, 127.3, 126.6, 125.5, 125.3, 100.4, 79.1, 70.1, 69.3, 47.1, 40.4, 28.4; HRMS (ESI) calcd for $C_{24}H_{28}N_6NaO_3$ $(M+Na)^+$ 471.2115. Found 471.2116.

Synthesis of 1NA-PP1-L1

1NA-PP1-L1B (32 mg, 71 μmol) was dissolved in trifluoroacetic acid (0.50 mL), stirred for 20 min at room temperature, and then evaporated to dryness. Aqueous sodium hydroxide was added to the residue, and extracted with ethyl acetate. The organic phase was washed with brine, dried over sodium sulfate, and evaporated to dryness, yielding 1NA-PP1-L1 (26 mg, quant).

^{1}H NMR (400 MHz, $CDCl_3$) δ 8.43 (s, 1 H), 8.00-7.92 (m, 3 H), 7.68-7.50 (m, 4 H), 5.02 (br, 2 H), 4.72 (t, J = 5.6, 2 H), 4.04 (t, J = 5.6, 2 H), 3.53 (t, J = 5.2, 2 H), 2.81 (t, J = 5.2, 2 H); ^{13}C NMR (125 MHz, $CDCl_3$): δ 157.6, 156.2, 154.6, 142.7, 133.9, 131.5, 130.0, 129.8, 128.6, 128.4, 127.3, 126.6, 125.5, 125.3, 100.3, 73.2, 68.9, 46.9, 41.8; HRMS (ESI) calcd for $C_{19}H_{21}N_6O$ $(M+H)^+$ 349.1771. Found 349.1765.

Synthesis of 1NA-PP1-L2B

1NA-PP1-L2B was similarly prepared from **2** (33 mg, 0.13 mmol) and **4** (30 mg, 84 μmol) according to the procedure described for 1NA-PP1-L1B, yielding 20 mg (44%).

^{1}H NMR (400 MHz, $CDCl_3$) δ 8.43 (s, 1 H), 8.02-7.93 (m, 3 H), 7.68-7.52 (m, 4 H), 5.13 (br, 1 H), 5.02 (br, 2 H), 4.72 (t, J = 6.0, 2 H), 4.07 (t, J = 6.0, 2 H), 3.68-3.46 (m, 10 H), 3.29-3.24 (m, 2 H), 1.43 (s, 9 H); ^{13}C NMR (125 MHz, $CDCl_3$): δ 157.7, 156.1, 156.0, 154.7, 142.7, 134.0, 131.6, 130.1, 129.8, 128.5, 128.3, 127.2, 126.6, 125.5, 125.4, 100.3, 79.1, 70.5 (4C), 70.2, 69.2, 46.8, 40.4, 28.4; HRMS (ESI) calcd for $C_{28}H_{36}N_6NaO_5$ $(M+Na)^+$ 559.2639. Found 559.2639.

Synthesis of 1NA-PP1-L2

1NA-PP1-L2 was similarly prepared from 1NA-PP1-L2B (12 mg, 0.13 mmol) according to the procedure described for 1NA-PP1-L1, yielding 9.1 mg (92%).

^{1}H NMR (400 MHz, $CDCl_3$) δ 8.40 (s, 1 H), 8.01-7.92 (m, 3 H), 7.68-7.51 (m, 4 H), 5.15 (br, 2 H), 4.72 (t, J = 6.0, 2 H), 4.05 (t, J = 6.0, 2 H), 3.69-3.52 (m, 8 H), 3.44 (t, J = 5.2, 2 H), 2.80 (t, J = 5.2, 2 H, 1.93 (br, 2H); ^{13}C NMR (125 MHz, $CDCl_3$): δ 157.6, 156.1, 154.6, 142.6, 133.9, 131.5, 130.0, 129.8, 128.5, 128.3, 127.3, 126.6, 125.5, 125.3, 100.3, 73.0, 70.47, 70.45, 70.4, 70.2, 69.2, 46.8, 41.6; HRMS (ESI) calcd for $C_{23}H_{29}N_6O_3$ $(M+H)^+$ 437.2296. Found 437.2309.

Supplementary Methods

Surface plasmon resonance assay

Setting 1NA-PP1-Lx and ASK1 KD as a ligand and an analyte, respectively, the SPR signal was measured with Biacore T100 (GE Healthcare). 1NA-PP1-L1 or 1NA-PP1-L2 was immobilized to a CM5 sensor chip. GST-tagged kinase domain recombinants of mouse wild-type ASK1 and as-ASK1 (V745L/M761A/S828A) were purified as previously described[21] and injected with a series of concentrations. The obtained sensorgrams were fitted to the bivalent analyte model because the analyte ASK1 KD can be dimerized in solution[20]. The dissociation constant for the first phase (K_{D1}) was calculated as the dissociation rate for the first phase (k_{d1}) divided by the association rate constant for the first phase (k_{a1}).

Gel filtration column chromatography

To fractionate the as-ASK1 signalosome in the brain, whole brains were isolated from six *Ask1*^{ASKA} knock-in mice and triturated with a Dounce homogenizer in H buffer (50 mM HEPES-KOH (pH 7.5), 10 mM KCl, 1 mM EDTA, 1 mM EGTA, 1.5 mM $MgCl_2$) containing 0.2% Triton X-100 and 150 mM NaCl. To fractionate the as-ASK1 signalosome in primary brown adipocytes, differentiated primary brown adipocytes were collected by scraping with H buffer containing 1% digitonin (Wako Pure Chemicals Industry, #044-02121) and homogenized with a pellet mixer (TreffLab). The homogenate was clarified by centrifugation, and the supernatant was filtered through a Millex-HV syringe filter unit (0.45 µm), PVDF (33 mm), and gamma sterilized (Millipore, #SLHV033RB) twice. The clarified homogenate was processed by gel filtration column chromatography using AKTA (GE Healthcare) with a Superose 6 10/300 GL column (GE Healthcare) and H buffer.

In Fig. 1g, the sample “after purification” was processed for the following section “purification of the as-ASK1 signalosome” prior to gel filtration column chromatography. For sampling for immunoblotting analysis, each fraction was precipitated using ethanol by incubation at −20 °C overnight and subsequent sedimentation through centrifugation. After air-drying for an hour, the samples were dissolved in SDS sample buffer (80 mM Tris-HCl pH 8.8, 80 µg/mL bromophenol blue, 28.8% glycerol, 4% SDS and 10 or 20 mM dithiothreitol).

Preparation of 1NA-PP1-Lx-immobilized beads

N-Hydroxysuccinimide (NHS) FG beads (TAMAGAWA SEIKI, #TAS8848 N1141) were sonicated in *N*,*N*-dimethylformamide (DMF; Sigma-Aldrich, #227056) several times and subsequently incubated with 130 µM 1NA-PP1-Lx for an hour at room temperature. The beads were shaken in 10% volume/volume ethanolamine (Sigma-Aldrich, #398136) in DMF at room temperature for 2 h, and the supernatant was replaced with 2 M Tris-HCl (pH 8.0). After sonication, the beads were shaken for another 2 h at room temperature. The medium was replaced with 10% volume/volume MeOH (Wako Pure Chemicals Industries, #137-01823) in H buffer, and the beads were sonicated. The prepared beads were stored at 4 °C. Prior to pull-down analysis, beads were sonicated again with H buffer and resuspended with H buffer.

Purification of the as-ASK1 signalosome

The fractionated samples or the clarified cell lysates of the as-ASK1 signalosome were incubated with 1NA-PP1-Lx-immoribilized beads for 16 h at 4 °C with gentle shaking, followed by addition of Washing H buffer (H buffer containing 0.2% Triton X-100). The beads were clarified by centrifugation followed by the addition of lysis buffer, and washed with Washing H buffer four times using a magnetic stand (TAMAGAWA SEIKI, #TA4899N10). The as-ASK1 complex was eluted from the beads by 1 mM 1NA-

PP1 or DMSO diluted in Washing H buffer for 2 h incubation on ice with tapping every 15 min. After the elution step, the supernatants were purified using a magnetic stand.

In Fig. 1f, for sampling for immunoblotting analysis, the supernatants or beads were sampled by adding SDS sample buffer.

Quantitative RT-PCR analysis

Total RNA was isolated from tissues or cells using Isogen (Nippon Gene, #319-90211) and reverse transcribed with ReverTra Ace qPCR RT Master Mix with gDNA Remover (Toyobo, #FSQ-301). Quantitative reverse transcription-PCR (RT-PCR) was carried out using a LightCycler 96 (Roche) or a QuantStudio 1 Real-Time PCR System (ABI) with SYBR Green PCR Master Mix (Kapa Biosystems, Cat#KK4602). Data were normalized to ribosomal protein S18 (*Rps18*). Primer sequences are listed in Supplementary Table S3.

Coimmunoprecipitation assay

The supernatants of cell extracts were incubated with anti-Flag-tag antibody-immobilized beads (Wako Pure Chemicals Industries, clone 1E6, #016-22784) for 10–30 min at 4 °C or incubated with anti-RIPK2 (#ab75257) antibody followed by the addition of Dynabeads Protein G (Invitrogen, #DB10004). The beads were washed with lysis buffer four times, followed by the direct addition of SDS sample buffer.

TUBE pull-down assay

The K63-linked polyubiquitin conjugates were purified using K63 TUBE-coupled magnetic beads (LifeSensors, #UM404M)) following the manufacturer's instructions. In brief, cells were lysed in lysis buffer, and the supernatants of the cell extracts were incubated with TUBE for 3 h at 4 °C. The beads were washed three times with chilled TBS-T. The samples were dissolved in SDS sample buffer.

Supplementary Figure

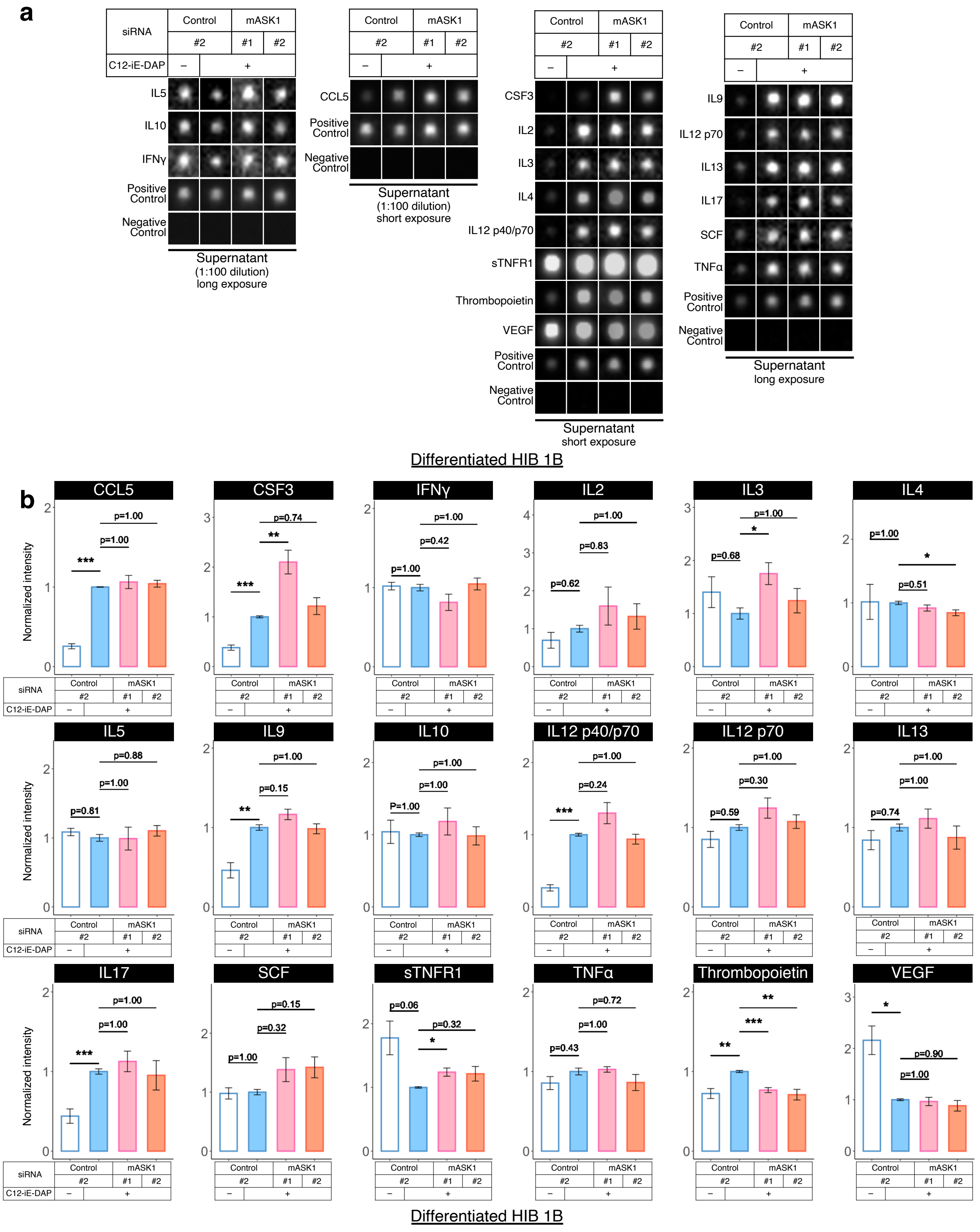

Supplementary Figure S1. Systematic analysis of the effects of ASK1 knockdown on cytokine secretion from NOD-RIPK2 pathway-activated cells.

(**a**) Supernatant from C12-iE-DAP (10 μg/mL, 8 h)-treated HIB 1B cells were subjected to cytokine antibody array. (**b**) The images of cytokine antibody array in (**a**) were quantified. Data are represented as the mean ± SEM. $n = 6$ (pooled from 3 independent experiments) for CCL5, IFNγ, IL5, IL10, and $n = 8$ (pooled from 4 independent experiments) for the other cytokines. $^{*}P < 0.05$, $^{**}P < 0.01$, $^{***}P < 0.001$ according to two-tailed Welch's test with the Bonferroni correction. Ctrl: control. See Fig. 3e,f for IL6, CCL2, CCL12 and CSF2.

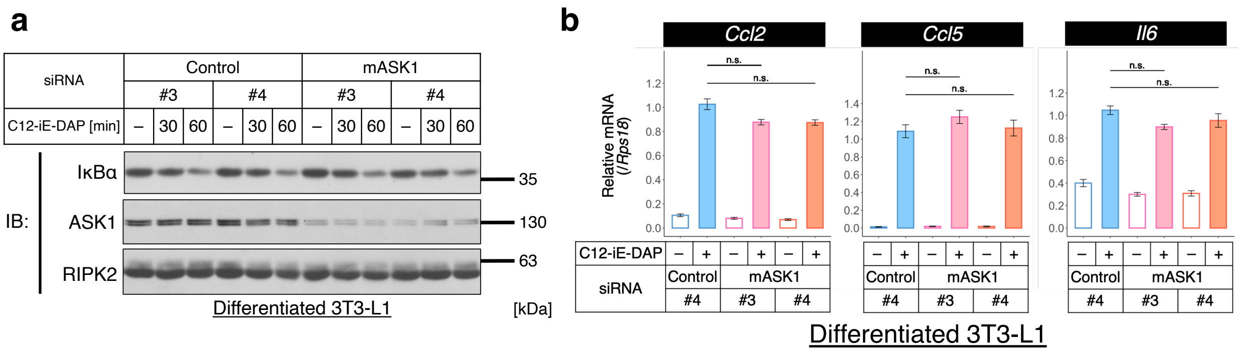

Supplementary Figure S2. ASK1 does not suppress the NOD-RIPK2 pathway and cytokine induction in white adipocytes.

(**a**) Effects of ASK1 knockdown on NOD-RIPK2 pathway activation in white adipocytes. 3T3-L1 cells were differentiated into white adipocytes and treated with C12-iE-DAP (10 µg/mL) for the indicated times. Note that cycloheximide (50 µg/ml) was treated to prevent the rapid feedback synthesis of IκBα. (**b**) Relative mRNA levels of inflammatory cytokines under NOD-RIPK2 pathway activation. 3T3-L1 cells were differentiated into white adipocytes and stimulated with C12-iE-DAP (10 µg/mL, 6 h). Data are represented as the mean ± SEM. n = 8–10 (pooled from 5 independent experiments). n.s.: not significant according to one-tailed Dunnett's test.

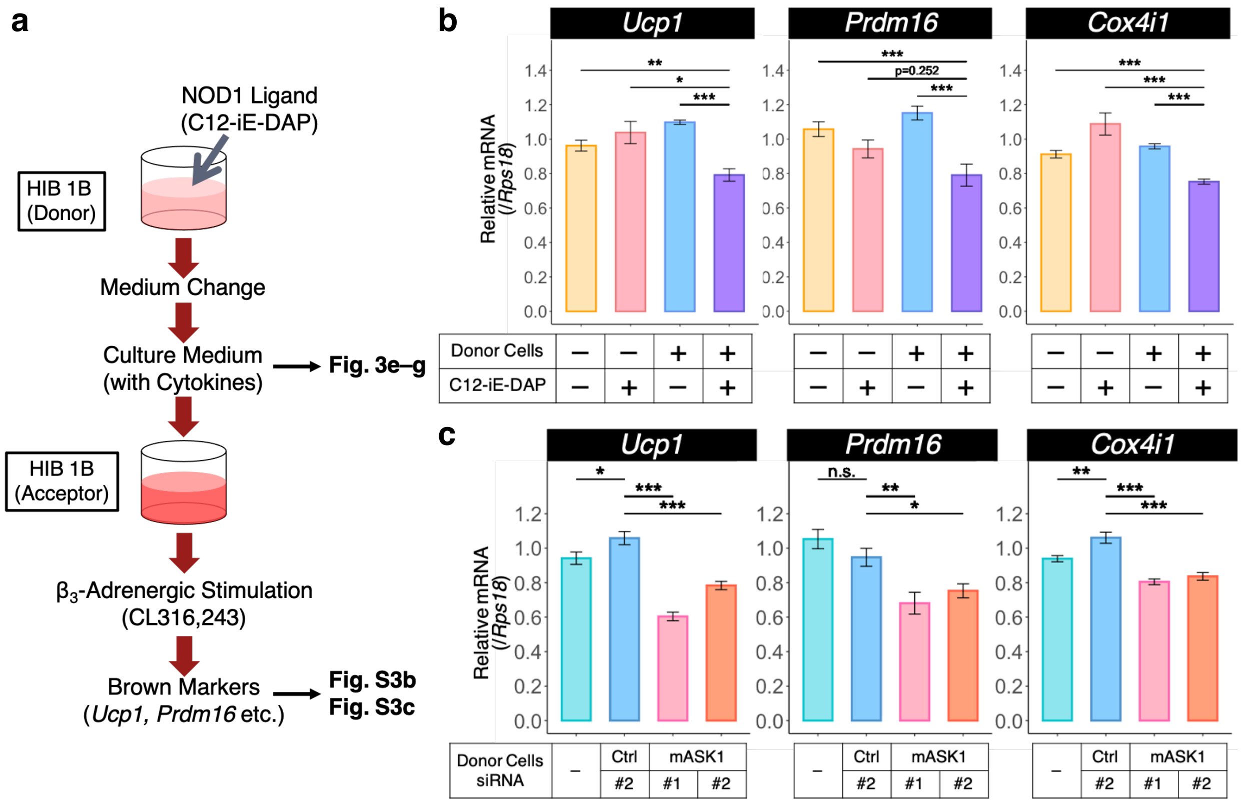

Supplementary Figure S3. Inhibition of the NOD-RIPK2 pathway contributes to maintenance of thermogenic potential in brown adipocytes.

(**a**) Experimental model for evaluating the paracrine effect of the NOD-RIPK2 pathway on brown adipocyte marker expression. Control or ASK1 knockdown HIB 1B cells (referred to as "donor cells") were treated with C12-iE-DAP. After changing the medium to avoid contamination of remnant C12-iE-DAP in the conditioned medium, the cells were incubated for 9 h in total. Then, the conditioned medium from the donor cells was used to treat another set of HIB 1B cells ("acceptor cells") for 6 h, followed by administration of 0.1 μM of a β_3-adrenergic receptor agonist CL316,243 for 12 h to induce the thermogenic genes in the acceptor cells. (**b**) Intercellular effects of the NOD-RIPK2 pathway on brown adipocyte marker induction. Acceptor HIB 1B cells were incubated with the conditioned medium from donor HIB 1B cells or donor cell-absent wells, with or without stimulation with C12-iE-DAP, and subsequently stimulated with CL316,243. Thermogenic marker induction in the acceptor cells was evaluated with quantitative RT-PCR. (**c**) Effects of ASK1 knockdown in NOD-RIPK2 pathway-activated donor cells on brown adipocyte marker induction in acceptor cells. Acceptor HIB 1B cells were incubated with the conditioned medium from ASK1-knockdown donor HIB 1B cells and subsequently stimulated with CL316,243. Thermogenic marker induction in the acceptor cells was evaluated with quantitative RT-PCR.

Data are represented as the mean ± SEM, $n = 9$ (pooled from 3 independent experiments) in (**b**), $n = 11$–12 (pooled from 3 independent experiments) in (**c**). *$P < 0.05$, **$P < 0.01$, ***$P < 0.001$. n.s.: not significant according to two-tailed Student's t-test with the Bonferroni correction (**b**) or two-tailed Dunnett's test (**c**).

Supplementary Table

Supplementary Table S1. Information on the antibodies used in this study.

Antibody	Source	Identifier
Anti-phospho-ASK1 polyclonal antibodies	Previous work[57]	N/A
Anti-phospho-p38 antibody (T180/Y182)	Cell Signaling Technology	#9211
Anti-phospho-p38 antibody (T180/Y182)	Cell Signaling Technology	#4511
Anti-phospho-JNK antibody (T183/Y185)	Cell Signaling Technology	#9251
Anti-p38α antibody	Cell Signaling Technology	#9228
Anti-ASK1 antibody	Abcam	#ab45178
Anti-RIPK2 antibody	Abcam	#ab75257
Anti-GFP antibody	MBL	#M048-3
Anti-HA-tag antibody	Roche	#11867431001
Anti-Flag-tag antibody	Wako Pure Chemicals Industries	#012-22384
Anti-Myc-tag antibody	Santa Cruz Biotechnology	#sc-40
Anti-JNK1 antibody	Santa Cruz Biotechnology	#sc-571
Anti-RIPK2 antibody	Santa Cruz Biotechnology	#sc-22763
Anti-IκBα antibody	Santa Cruz Biotechnology	#sc-371
Anti-IL-6 antibody	Santa Cruz Biotechnology	#sc-57315
HRP-linked anti-rabbit IgG	Cell Signaling Technology	#7074
HRP-linked anti-mouse IgG	Cell Signaling Technology	#7076
HRP-linked anti-rat IgG	Cell Signaling Technology	#7077
HRP-linked anti-protein G	SouthernBiotech	#7506-05

Supplementary Table S2. Information on the siRNAs used in this study.

siRNA	Source	Identifier
Control siRNA #1 (Stealth RNAi Negative Control Medium GC Duplex #1)	Invitrogen	#12935-112
Control siRNA #2 (Stealth RNAi Negative Control Medium GC Duplex #2)	Invitrogen	#12935-113
Human ASK1 siRNA #1 (Stealth RNAi siRNA, target sequence: 5′-GCCAACACUACAGUCAGGAAUUAAU-3′)	Invitrogen	#10620312, #1278
Human ASK1 siRNA #2 (Stealth RNAi siRNA, target sequence: 5′-UGAAGCUAAGUAGUCUUCUUGGUAA-3′)	Invitrogen	#10620312, #1370
Human ASK1 siRNA #3 (Stealth RNAi siRNA, target sequence: 5′-CCUGUGCUAACGACUUGCUUGUUGA-3′)	Invitrogen	#10620312, #2780
Mouse ASK1 siRNA #1 (Stealth RNAi siRNA, target sequence: 5′-AAUUGCAGUGUGCACAGCCUUUCGG-3′)	Invitrogen	#10620312
Mouse ASK1 siRNA #2 (Stealth RNAi siRNA, target sequence: 5′-AAAUGCGUAAUGAAACUUCACGUGG-3′)	Invitrogen	#10620312
Mouse RIPK2 siRNA (Stealth RNAi siRNA, target sequence: 5′-GGGCCAGUGUGAAGCAUGAUAUAUA-3′)	Invitrogen	#10620312
Control siRNA #3 (ON-TARGET*plus* Non-targeting siRNA #3)	Dharmacon	#D-001810-03
Control siRNA #4 (ON-TARGET*plus* Non-targeting siRNA #4)	Dharmacon	#D-001810-04
Mouse ASK1 siRNA #3 (ON-TARGET*plus* siRNA, target sequence: 5′-GCUUAUUGAUGAGUUCUUA-3′)	Dharmacon	#J-041179-07
Mouse ASK1 siRNA #4 (ON-TARGET*plus* siRNA, target sequence: 5′-GAAACUAAUAGGUUACUUG-3′)	Dharmacon	#J-041179-08

Supplementary Table S3. Sequences of quantitative RT-PCR primers used in this study.

Gene	Forward	Reverse
Ccl2	cactcacctgctgctactca	gcttggtgacaaaaactacagc
Ccl5	tgcagcggactctgacacagc	gagtggtgtccgagccata
Cxcl2	aaaatcatccaaaagatactgaacaa	ctttggttcttccgttgagg
Il6	gctaccaaactggatataatcagga	ccaggtagctatggtactccagaa
Tnfa	ctgtagcccacgtcgtagc	ttgagatccatgccgttg
Ucp1	gatgtggtaaaaacaagattcatca	cgcagaaaagaagccacaa
Prdm16	tctcggatcccatcctca	ggaagatcttgccacagtacct
Cox4i1	tcactgcgctcgttctgat	cgatcgaaagtatgagggatg
Rps18	acttttggggccttcgtgtc	gcaaaggcccagagactcat
